# Incidence and relative risk of metachronous second primary cancers for 16 cancer sites, Osaka, Japan, 2000–2015: Population‐based analysis

**DOI:** 10.1002/cam4.4457

**Published:** 2021-11-29

**Authors:** Satomi Odani, Takahiro Tabuchi, Kayo Nakata, Toshitaka Morishima, Yoshihiro Kuwabara, Shihoko Koyama, Haruka Kudo, Mizuki Kato, Isao Miyashiro

**Affiliations:** ^1^ Cancer Control Center Osaka International Cancer Institute Osaka Japan; ^2^ Division of Cancer Medicine Graduate School of Medicine Osaka University Osaka Japan

**Keywords:** alcohol‐related cancer, cumulative risk, epidemiology, incidence rate, multiple primary cancers, smoking‐related cancer, standardized incidence ratio

## Abstract

**Background:**

An increasing number of cancer survivors have developed multiple primaries. This study aims to describe the incidence and risk patterns of metachronous second primary cancers (SPCs) in Osaka, Japan.

**Methods:**

Data were obtained from the Osaka Cancer Registry, a population‐based database of all cancers diagnosed in Osaka. The study subjects were individuals who were first diagnosed with invasive cancers in 16 major cancer sites during 2000–2014, aged 15–79 years, survived at least 3 months, and were followed up for 10 years. We measured incidence rates, cumulative risks, and standardized incidence ratios (SIRs: with the Osaka general population as the referent) of developing SPCs during 3 months to 10 years after the first diagnosis.

**Results:**

During 2000–2015, among 418,791 cancer survivors, 24,368 (5.8%) developed SPCs within 10 years of first diagnosis. Males had higher incidence rates than females except among young‐onset survivors (aged 15–39 years). 10‐year cumulative risks among survivors aged 70–79 years (the most dominant age group) were 24.0% (male) and 11.8% (female). 10‐year SIRs were 1.38 (95% CI, 1.36–1.40; male) and 1.44 (95% CI, 1.41–1.48; female) with higher estimates among younger survivors in both sexes. Strong bidirectional associations were observed between oral/pharyngeal, esophageal, and laryngeal cancers. Survivors of any smoking‐related cancers had elevated SIRs of developing smoking‐related SPCs. Similar results were observed for alcohol‐related cancers.

**Conclusions:**

Cancer survivors are at excess risk of developing SPCs compared to the general population. Continued surveillance is warranted to inform survivorship care through risk‐based long‐term care planning and lifestyle‐changing efforts to prevent new cancers.

## Graphical abstract

During 2000–2015, 5.8% of cancer survivors in Osaka, Japan, developed metachronous second primary cancers (SPCs) within 10 years of first diagnosis. Cancer survivors were at 1.38 times (male) and 1.44 times (female) higher risks of developing SPCs compared to the general population. Coordinated efforts of survivorship care through risk‐based care planning, surveillance, and lifestyle‐changing interventions are warranted.

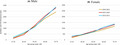

## 1 INTRODUCTION

Developing multiple primary cancers has become a common rather than rare event that patients and clinicians encounter.^1^, ^2^, ^3^, ^4^ Of all cancer survivors, the incidence of multiple primaries is reported to be in the range of 2%–17%.^1^, ^2^, ^3^, ^4^, ^5^ In our previous assessments in Osaka, Japan, 2.0% of cancer survivors developed a second primary cancer within 10 years of first diagnosis during 1966–1989,^6^ and this proportion rose nearly double (3.8%) by 1985–2005.^4^ It is assumed that there has been a further increase to date given the improved detection techniques, prolonged survival, and aging of survivors in the past decade.^7^

Several studies in high‐income countries have reported that cancer survivors are at higher risk of subsequent diagnosis with another cancer relative to the general population.^2^, ^3^, ^4^, ^11^ While the extent of such associations varies depending on the types of the first and second cancers, strong associations are seen between lifestyle‐related (e.g. smoking and alcohol consumption) cancers.^2^, ^4^, ^6^, ^9^, ^10^, ^11^, ^12^ Our previous report contributed to this evidence base by presenting site‐specific risk patterns: survivors of oral/pharyngeal, esophageal, and laryngeal cancers had 4–22 times higher risks of developing a second primary in either of these three sites compared to the general population.^4^ As a decade has passed since our previous assessment, there is a need for more recent, comprehensive, and implicative data that reflect current landscape of cancer care.

Given the growing presence of multiple primary cancers, understanding its etiology and trend is key to informing clinicians to practice risk‐based care, promoting preventive behaviors among survivors, and guiding policy makers to decide the future directions and priorities of survivorship care. This study aims to describe the latest incidence and risk patterns of second primary cancers using data from the Osaka Cancer Registry—one of the largest population‐based cancer registries in the world. This is an update to our previous reports since the 1960s.^4^, ^6^

## 2 MATERIALS AND METHODS

### 2.1 Data and study cohort

For the present retrospective cohort study, data were obtained from the Osaka Cancer Registry (OCR), a population‐based database of all cancers in Osaka prefecture, Japan (population 8.9 million as of 2010).^13^, ^14^ The OCR is one of the few longitudinal cancer databases of Japan that meet the international qualifications of comparability, validity, timeliness, and completeness.^15^ Since it was founded in 1962, the OCR has gathered reports from medical facilities and the death certificate database and followed up vital status of each report until 10 years from the diagnosis. The study subjects for the present study comprised all individuals who were first diagnosed with any invasive primary cancer (first primary cancer, FPC hereinafter) during 2000–2014, aged 15–79 years and resided in Osaka at the time of diagnosis. Registrations that were notified by death certificate only (DCO) were excluded from the analysis (5.8% of all registrations). This study was approved by the Institutional Review Board of Osaka International Cancer Institute (approval number: 18–0018). We obtained the dataset with no personally identifiable information from the OCR, and independently processed it in accordance with the Act on Promotion of Cancer Registries.^13^

### 2.2 Definition of metachronous second primary cancers (SPCs)

A metachronous second primary cancer (referred to as SPC hereinafter) was defined as a subsequent primary cancer that occurred during 3 months to 10 years after diagnosis of the FPC.^4^, ^6^ We used the suggested rules of the International Association for Research on Cancer (IARC)^16^ and the third edition of the ICD‐O^17^ to identify individuals with multiple primary cancers. Under the IARC’s definition, one tumor should only be recognized in an organ, a pair of organs, or tissues.^16^ For example, with a stomach cancer as the FPC, subsequent stomach cancer is not counted as an SPC unless its histological type differs from that of the FPC; thus, this leads to apparently low estimates of SPC incidence, especially after common cancers such as colorectum, prostate and female breast cancers. To reduce such potential underestimation and keep consistency to our previous reports,^4^, ^6^ we excluded cancers that occurred in the same site as the FPC from the analyses regardless of their histological types (see next section for details).

Cancer sites were classified into 16 major categories according to the International Classification of Diseases Tenth Revision (ICD‐10)^18^ as follows: oral cavity/pharynx (C00–14), esophagus (C15), stomach (C16), colorectum (C18–20), liver (C22), gallbladder (C23, C24), pancreas (C25), larynx (C32), lung (C33, C34), breast (female) (C50), uterus (C53–55), ovary (C56), prostate (C61), kidney/urinary tract/bladder (C64–68), thyroid (C73), and blood (C81–85, C88, C90, C91–96). In situ carcinomas, benign intracranial tumors, male breast cancers, and any third or later primaries were not included in the analysis.

### 2.3 Statistical analysis

To estimate the risks of developing SPCs, person‐years at risk were calculated as the time from 3 months after diagnosis of the FPC until whichever of the following came first: (i) December 31, 2015, (ii) diagnosis of an SPC, (iii) death, or (iv) 10 years after the FPC diagnosis. Incidence rates and cumulative risks were examined by sex, age, and calendar year of the FPC diagnosis or duration of follow‐up.

To compare the risk of cancer survivors developing an SPC to the risk of the general population developing a cancer, standardized incidence ratio (SIR) was computed as a ratio of the observed number to the expected number of SPCs. Using 26 sex‐age strata (male and female in 5‐year age strata), the observed numbers of SPCs were counted within each stratum. The expected numbers of SPCs were also calculated within each sex‐age stratum by multiplying the person‐years at risk and the cancer incidence rates of the Osaka general population. Both expected and observed numbers were then weighted and summed to be proportional to the size of the corresponding sex‐age subgroups of the general population to account for the differences in sex‐age distributions between cancer survivors and the general population. To avoid the underestimation of site‐specific SIRs (mentioned in the previous subsection), we did not count the cancers that occurred in the same site as the FPC for calculating both the observed and expected numbers of SPCs. SIRs were then obtained by dividing the observed numbers by the expected numbers for each of the assessed cancer sites and all sites combined. 95% confidence intervals (95% CIs) were calculated assuming a Poisson distribution.^19^ We further assessed SIRs of developing smoking‐related SPCs among survivors of smoking‐related cancers (oral/pharyngeal, esophageal, stomach, liver, pancreatic, laryngeal, lung, and kidney/urinary tract/bladder cancers).^20^, ^21^, ^22^ Similar analyses were performed for six alcohol‐related cancers (oral/pharyngeal, esophageal, colorectal, liver, laryngeal, and breast cancers).^21^, ^23^, ^24^ All analyses were conducted using R version 4.0.3.

## 3 RESULTS

**FIGURE 1:**
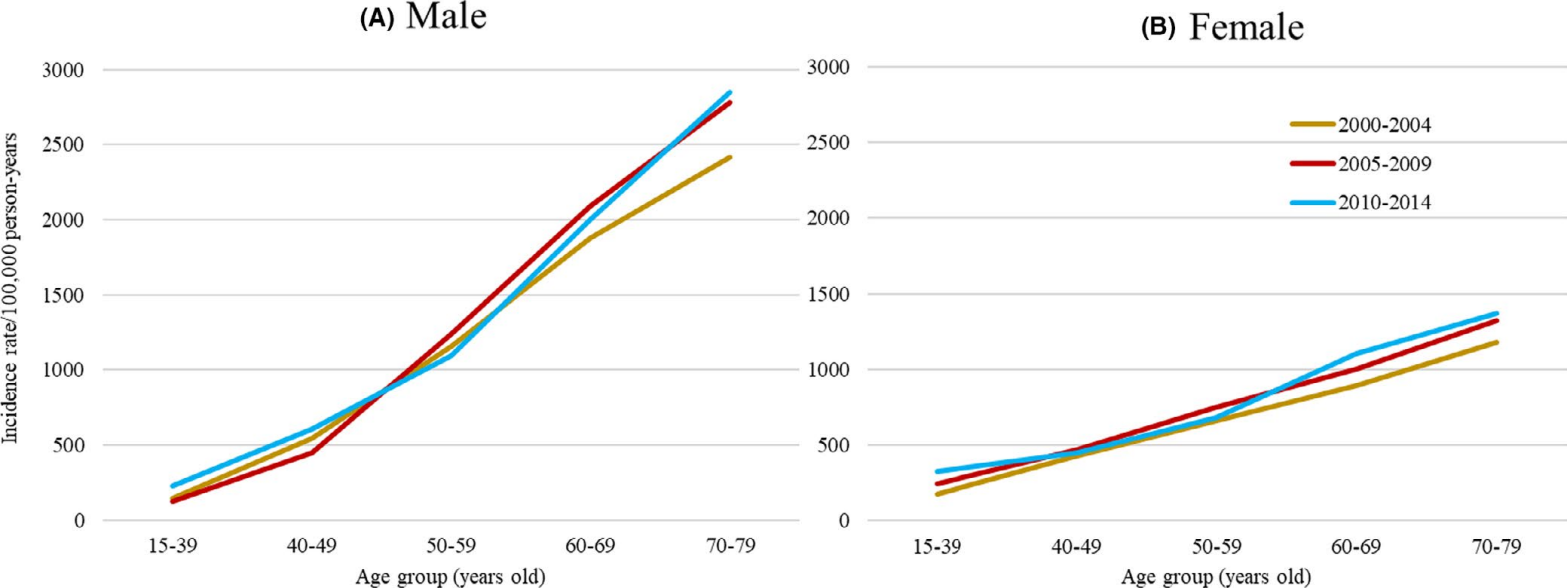
Age‐specific incidence rates of metachronous second primary cancers per 100,000 person‐years by sex and calendar year of diagnosis of the first primary cancer, Osaka Cancer Registry, Japan, 2000–2015. Note: A metachronous second primary cancer was defined as a subsequent primary cancer that occurred during 3 months to 10 years after diagnosis of the first primary cancer. Person‐years at risk were calculated as the time from 3 months after diagnosis of the first primary cancer until whichever of the following came first: (i) December 31st, 2015, (ii) diagnosis of an SPC, (iii) death, or (iv) 10 years after the FPC diagnosis

During 2000–2015, of a total of 418,791 cancer survivors (mean age 64.1 years; 57.9% male; 87.1% histologically confirmed), 24,368 (5.8%) developed an SPC after a median of 2.84 years (mean 3.97 years). Figure 1 presents age‐ and sex‐specific incidence rates of SPCs per 100,000 person‐years by calendar year of first diagnosis. While incidence rates increased with age and were generally higher among males, females had higher incidence rates than males in the youngest group aged 15–39 years.

Table 1 demonstrates cumulative risks of developing SPCs by sex, age, and calendar year of the FPC diagnosis. Cumulative risks increased with age and were generally higher among males than females except in the youngest group aged 15–39 years. Throughout the study period, 5‐year cumulative risks ranged from 0.7% (aged 15–39 years) to 12.1% (aged 70–79 years) in males and 1.2% (aged 15–39 years) to 6.1% (aged 70–79 years) in females: 10‐year cumulative risks ranged from 1.3% (aged 15–39 years) to 24.0% (aged 70–79 years) in males and 2.2% (aged 15–39 years) to 11.8% (aged 70–79 years) in females. When stratified by calendar year of first diagnosis, cumulative risks generally increased over time. Among survivors aged 70–79 years (the most dominant age group), 5‐year cumulative risk increased from 10.4% (2000–2004) to 12.8% (2010–2014) in males; and 5.4% (2000–2004) to 6.2% (2010–2014) in females.

**TABLE 1:** Cumulative risk of developing metachronous second primary cancers (%) by sex, age, and calendar year at diagnosis of the first primary cancer, Osaka Cancer Registry, Japan, 2000–2015

		Calendar year at diagnosis of the first primary cancer			
Sex/age (years)	Time since diagnosis of the first primary cancer	2000–2004	2005–2009	2010–2014	Overall (2000–2014)
Male					
15–39	3 months–5 years	0.8	0.6	0.7	0.7
	3 months–10 years	1.4	1.1	—	1.3
40–49	3 months–5 years	2.3	1.9	2.9	2.3
	3 months–10 years	5.4	4.1	—	5.1
50–59	3 months–5 years	4.3	5.3	5.4	5.0
	3 months–10 years	11.4	12.4	—	12.0
60–69	3 months–5 years	7.5	9.0	9.4	8.7
	3 months–10 years	17.9	19.6	—	19.2
70–79	3 months–5 years	10.4	12.3	12.8	12.1
	3 months–10 years	22.0	24.7	—	24.0
Female					
15–39	3 months–5 years	0.8	1.3	1.2	1.2
	3 months–10 years	1.8	2.3	—	2.2
40–49	3 months–5 years	1.5	1.9	2.3	1.9
	3 months–10 years	4.2	4.8	—	4.6
50–59	3 months–5 years	2.8	3.3	3.3	3.1
	3 months–10 years	6.4	7.0	—	6.8
60–69	3 months–5 years	3.7	4.3	5.0	4.4
	3 months–10 years	8.7	9.9	—	9.7
70–79	3 months–5 years	5.4	6.3	6.2	6.1
	3 months–10 years	11.1	11.9	—	11.8
All					
15–39	3 months–5 years	0.8	1.2	1.1	1.1
	3 months–10 years	1.7	2.0	—	2.0
40–49	3 months–5 years	1.7	1.9	2.5	2.0
	3 months–10 years	4.5	4.6	—	4.8
50–59	3 months–5 years	3.5	4.2	4.2	4.0
	3 months–10 years	8.7	9.5	—	9.2
60–69	3 months–5 years	6.0	7.1	7.7	7.0
	3 months–10 years	14.1	15.7	—	15.3
70–79	3 months–5 years	8.4	10.1	10.5	9.9
	3 months–10 years	17.5	19.8	—	19.2

Note

A metachronous second primary cancer was defined as a subsequent primary cancer that occurred during 3 months to 10 years after diagnosis of the first primary cancer. Patients were followed up until whichever of the following came first: diagnosis of metachronous second primary cancers; December 31st, 2015; or death.

Table 2 shows relative risks of developing SPCs by sex, age, and follow‐up intervals. Throughout the 10‐year follow‐up, there were 17,552 SPCs with 876,528 person‐years among males overall, which corresponded with a 38% higher risk of developing SPCs relative to the risk in the general population (SIR = 1.38 [95% CI, 1.36–1.40]). Among females overall, there were 6,816 SPCs with 785,595 person‐years which corresponded with a 44% higher risk of developing SPCs compared to the general population (SIR = 1.44 [95% CI, 1.41–1.48]). 10‐year SIRs were highest among the youngest survivors aged 15–39 years and attenuated with increasing age. The youngest group had 5.66 (95% CI, 3.93–7.99) and 2.80 (95% CI, 2.38–3.26) times higher risks of developing SPCs in males and females, respectively, compared to the general population. With regard to the shift in SIRs over the 10‐year follow‐up, patterns differed according to survivor's age. Among survivors aged 15–39 years, SIR was highest within the first 1 year of the FPC diagnosis (15.52 [95% CI, 8.75–26.50] and 3.23 [95% CI, 2.09–4.69] for males and females, respectively). In other age groups generally, SIRs were highest during 5–10 years after the FPC diagnosis in both sexes.

**TABLE 2:** Observed numbers and standardized incidence ratios of developing metachronous second primary cancers by sex, age, and time since diagnosis of the first primary cancer, Osaka Cancer Registry, Japan, 2000–2015

						Time since diagnosis of the first primary cancer											
		3 months–1 year				1–5 years				5–10 years				Total (3 months–10 years)			
Age at FPC diagnosis (years)	Number of patients	Observed SPC, No	Person‐years	SIR	95% CI	Observed SPC, No	Person‐years	SIR	95% CI	Observed SPC, No	Person‐years	SIR	95% CI	Observed SPC, No	Person‐years	SIR	95% CI
Male																	
15–39	3615	11	2545	15.52	8.75–26.50	9	9111	3.63	1.96–6.56	7	5892	4.46	1.95–8.16	27	17548	5.66	3.93–7.99
40–49	9236	38	6447	4.41	3.15–5.98	98	21336	3.42	2.77–4.14	73	12363	4.36	3.42–5.45	209	40146	3.86	3.37–4.43
50–59	37538	271	25665	1.96	1.74–2.20	868	83436	1.93	1.81–2.06	731	48901	2.80	2.60–3.00	1870	158003	2.20	2.10–2.30
60–69	92989	1157	63159	1.36	1.28–1.44	3622	192223	1.40	1.36–1.45	2111	88864	1.79	1.71–1.87	6890	344246	1.50	1.46–1.53
70–79	99119	1761	65988	1.08	1.03–1.13	4864	183444	1.07	1.05–1.11	1931	66883	1.20	1.15–1.25	8556	316315	1.10	1.08–1.13
All (15–79)	242497	3238	163804	1.28	1.23–1.32	9461	489550	1.30	1.27–1.32	4853	222903	1.67	1.62–1.72	17552	876258	1.38	1.36–1.40
Female																	
15–39	10261	21	7576	3.23	2.09–4.69	76	30207	2.95	2.38–3.62	41	19891	2.41	1.78–3.18	138	57674	2.80	2.38–3.26
40–49	21665	76	15956	1.72	1.35–2.13	236	60586	1.41	1.23–1.59	197	36373	1.96	1.70–2.26	509	112914	1.63	1.49–1.78
50–59	35655	180	25648	1.60	1.38–1.84	620	95403	1.47	1.36–1.59	479	61364	1.78	1.62–1.94	1279	182415	1.59	1.51–1.68
60–69	54346	391	38323	1.50	1.35–1.65	1204	129841	1.37	1.29–1.45	747	67704	1.63	1.52–1.75	2342	235868	1.46	1.40–1.52
70–79	54354	495	36977	1.28	1.17–1.40	1438	110732	1.25	1.19–1.32	615	49015	1.22	1.13–1.32	2548	196724	1.25	1.20–1.30
All (15–79)	176281	1163	124480	1.46	1.37–1.54	3574	426769	1.37	1.32–1.41	2079	234347	1.58	1.51–1.65	6816	785595	1.44	1.41–1.48
All																	
15–39	13876	32	10121	4.42	3.19–6.10	85	39318	3.01	2.44–3.64	48	25783	2.59	1.95–3.34	165	75222	3.05	2.64–3.51
40–49	30901	114	22402	2.15	1.77–2.57	334	81922	1.70	1.52–1.88	270	48736	2.30	2.03–2.58	718	153060	1.95	1.81–2.10
50–59	73194	451	51315	1.79	1.63–1.96	1488	178842	1.71	1.62–1.80	1210	110265	2.27	2.15–2.40	3149	340421	1.90	1.84–1.97
60–69	147344	1548	101488	1.39	1.33–1.47	4826	322081	1.39	1.35–1.43	2858	156571	1.74	1.68–1.81	9232	580140	1.49	1.46–1.52
70–79	153476	2256	102968	1.12	1.07–1.17	6302	294182	1.12	1.09–1.14	2546	115898	1.21	1.16–1.25	11104	513047	1.14	1.12–1.16
All (15–79)	418791	4401	288294	1.32	1.28–1.37	13035	916345	1.32	1.29–1.34	6932	457253	1.64	1.60–1.68	24368	1661890	1.40	1.38–1.42

Note:

A metachronous second primary cancer was defined as a subsequent primary cancer that occurred during 3 months to 10 years after diagnosis of the first primary cancer. Person‐years at risk were calculated as the time from 3 months after diagnosis of the first primary cancer until whichever of the following came first: (i) December 31st, 2015, (ii) diagnosis of an SPC, (iii) death, or (iv) 10 years after the FPC diagnosis. SIRs were calculated as the ratio of the observed number to the expected number of second primary cancers to compare the risk of developing an SPC to the general population.

Abbreviations: CI, confidence interval; FPC, first primary cancer; SIR, standardized incidence ratio; SPC, second primary cancer.

Table 3 presents SIRs according to the FPC site. Elevated SIRs were observed for all the assessed sites except pancreas. SIRs were especially high among survivors of oral/pharyngeal, esophageal, and laryngeal cancers (2.98 [95% CI, 2.79–3.19], 2.82 [95% CI, 2.64–3.01], and 2.87 [95% CI, 2.64–3.12] in males; 2.42 [95% CI, 2.08–2.79], 3.22 [95% CI, 2.71–3.79], and 2.55 [95% CI, 1.61–3.75] in females, respectively). With regard to the shift in SIRs over the 10‐year follow‐up, SIRs were highest during 5–10 years after the first diagnosis for all the assessed FPC sites except thyroid in males; and except oral/pharynx, stomach, colorectum, ovary, and thyroid in females.

**TABLE 3:** Observed numbers and standardized incidence ratios of developing a second primary cancer by sex, site, and time since diagnosis of the first primary cancer, Osaka Cancer Registry, Japan, 2000–2015

							Time since diagnosis of the first primary cancer											
			3 months–1 year				1–5 years				5–10 years				Total (3 months–10 years)			
FPC site	ICD−10	Number of patients	Observed SPCs, No	Person‐years	SIR	95% CI	Observed SPCs, No	Person‐years	SIR	95% CI	Observed SPCs, No	Person‐years	SIR	95% CI	Observed SPCs, No	Person‐years	SIR	95% CI
Male																		
Oral cavity/ pharynx	C00–14	7508	187	5157	3.07	2.65–3.56	461	15189	2.84	2.59–3.12	213	7131	3.25	2.83–3.70	861	27478	2.98	2.79–3.19
Esophagus	C15	10859	181	6694	2.04	1.75–2.37	504	14623	2.70	2.47–2.95	261	5386	4.26	3.76–4.80	946	26703	2.82	2.64–3.01
Stomach	C16	49752	638	33817	1.53	1.40–1.66	2040	106643	1.64	1.56–1.71	1077	56350	1.89	1.78–2.01	3755	196810	1.69	1.63–1.74
Colorectum	C18–20	40822	500	29146	1.36	1.24–1.50	1709	97420	1.43	1.36–1.51	978	48289	1.88	1.76–2.00	3187	174855	1.54	1.48–1.59
Liver	C22	21469	272	14140	1.31	1.15–1.49	700	39159	1.26	1.16–1.36	247	12310	1.63	1.43–1.85	1219	65609	1.33	1.25–1.42
Gallbladder	C23, C24	4045	42	2388	1.16	0.84–1.59	90	4701	1.24	0.99–1.55	36	1579	1.69	1.19–2.36	168	8668	1.29	1.09–1.51
Pancreas	C25	7226	43	3734	0.79	0.58–1.10	60	4280	1.04	0.79–1.35	18	957	1.67	1.02–2.61	121	8971	0.99	0.82–1.19
Larynx	C32	3231	95	2334	2.89	2.33–3.54	301	8290	2.65	2.35–2.98	176	4360	3.31	2.84–3.84	572	14985	2.87	2.64–3.12
Lung	C33, C34	36235	363	22215	1.22	1.09–1.36	747	45225	1.32	1.22–1.43	362	15941	2.10	1.89–2.34	1472	83382	1.43	1.36–1.51
Prostate	C61	31391	437	23342	1.12	1.01–1.24	1589	85390	1.14	1.08–1.21	793	36591	1.41	1.31–1.52	2819	145323	1.21	1.16–1.26
Kidney/urinary tract/bladder	C64–68	16013	327	11322	2.10	1.86–2.35	827	38978	1.58	1.47–1.70	494	20068	2.14	1.95–2.34	1648	70367	1.82	1.73–1.91
Thyroid	C73	1811	25	1315	1.90	1.19–2.77	74	5070	1.64	1.29–2.07	38	2894	1.67	1.20–2.30	137	9279	1.69	1.42–2.00
Blood	C81–85, C88, C90, C91–96	11892	126	8023	1.29	1.05–1.54	347	23937	1.33	1.19–1.48	152	10701	1.71	1.45–2.00	625	42662	1.40	1.29–1.52
Female																		
Oral cavity/ pharynx	C00–14	2816	37	2007	3.02	2.16–4.14	96	6774	2.33	1.89–2.85	50	3937	2.24	1.68–2.93	183	12719	2.42	2.08–2.79
Esophagus	C15	2064	19	1341	2.16	1.35–3.29	71	3555	2.99	2.36–3.76	46	1547	4.69	3.47–6.13	136	6442	3.22	2.71–3.79
Stomach	C16	20454	176	14081	1.99	1.70–2.31	485	46148	1.66	1.51–1.82	269	26741	1.71	1.51–1.93	930	86970	1.73	1.62–1.85
Colorectum	C18–20	27635	226	19820	1.88	1.64–2.15	616	68158	1.54	1.42–1.67	339	37023	1.61	1.44–1.79	1181	125001	1.61	1.52–1.71
Liver	C22	9089	79	6110	1.61	1.27–2.03	208	17486	1.51	1.30–1.74	61	5272	1.65	1.27–2.12	348	28868	1.56	1.39–1.74
Gallbladder	C23, C24	3300	17	1903	1.16	0.68–1.84	35	3735	1.30	0.90–1.78	27	1399	2.72	1.84–3.94	79	7036	1.54	1.22–1.91
Pancreas	C25	5386	21	2839	1.02	0.63–1.54	30	3456	1.25	0.84–1.75	10	787	1.86	0.95–3.38	61	7083	1.22	0.93–1.56
Larynx	C32	228	1	168	0.90	0.21–4.76	12	660	2.75	1.52–4.61	8	422	2.93	1.45–5.56	21	1251	2.55	1.61–3.75
Lung	C33, C34	15975	98	10676	1.41	1.14–1.71	227	28043	1.23	1.07–1.40	111	11601	1.55	1.28–1.86	436	50320	1.34	1.21–1.47
Breast	C50	47622	231	35631	1.64	1.44–1.86	947	143472	1.72	1.61–1.83	665	87582	2.13	1.98–2.30	1843	266685	1.84	1.76–1.92
Uterus	C53–55	15839	85	11538	1.64	1.32–2.03	300	42337	1.69	1.51–1.89	199	24738	2.08	1.81–2.38	584	78613	1.80	1.66–1.95
Ovary	C56	6039	37	4289	1.77	1.28–2.43	94	13659	1.51	1.23–1.84	43	6753	1.59	1.17–2.10	174	24701	1.57	1.35–1.82
Kidney/urinary tract/bladder	C64–68	5117	43	3615	1.71	1.25–2.30	170	12327	1.94	1.65–2.25	98	6632	2.25	1.84–2.75	311	22574	1.99	1.77–2.23
Thyroid	C73	5336	49	3963	2.34	1.73–3.06	132	16176	1.61	1.35–1.90	73	9800	1.53	1.21–1.92	254	29939	1.68	1.49–1.90
Blood	C81–85, C88, C90, C91–96	9381	44	6499	1.13	0.83–1.51	151	20783	1.23	1.04–1.45	80	10114	1.54	1.23–1.90	275	37395	1.29	1.14–1.45
All																		
Oral cavity/pharynx	C00–14	10324	224	7175	3.06	2.67–3.50	557	22006	2.74	2.51–2.98	263	11093	2.98	2.64–3.37	1044	40274	2.86	2.69–3.04
Esophagus	C15	12923	200	8035	2.05	1.77–2.36	575	18177	2.74	2.51–2.97	307	6933	4.32	3.86–4.83	1082	33145	2.87	2.70–3.05
Stomach	C16	70212	814	47906	1.62	1.50–1.74	2525	152820	1.64	1.57–1.71	1346	83109	1.85	1.75–1.96	4685	283834	1.70	1.65–1.75
Colorectum	C18–20	68460	726	48972	1.50	1.39–1.62	2325	165596	1.46	1.40–1.52	1317	85316	1.79	1.70–1.90	4368	299884	1.56	1.51–1.61
Liver	C22	30558	351	20293	1.37	1.22–1.54	908	56809	1.31	1.22–1.40	308	17694	1.63	1.45–1.83	1567	94796	1.38	1.31–1.46
Gallbladder	C23, C24	7345	59	4291	1.16	0.88–1.51	125	8435	1.26	1.04–1.52	63	2978	2.05	1.57–2.62	247	15704	1.37	1.20–1.56
Pancreas	C25	12612	64	6582	0.86	0.66–1.11	90	7767	1.11	0.88–1.36	28	1766	1.73	1.16–2.48	182	16115	1.07	0.91–1.24
Larynx	C32	3459	96	2502	2.81	2.26–3.45	313	8951	2.66	2.36–2.98	184	4782	3.29	2.82–3.80	593	16236	2.85	2.62–3.10
Lung	C33, C34	52213	461	32896	1.26	1.14–1.39	974	73281	1.30	1.21–1.39	473	27550	1.93	1.76–2.12	1908	133726	1.41	1.34–1.48
Kidney/urinary tract/bladder	C64–68	21130	370	14985	2.04	1.82–2.27	997	51513	1.63	1.53–1.75	592	26883	2.16	1.98–2.34	1959	93381	1.85	1.76–1.93
Thyroid	C73	7147	74	5290	2.18	1.72–2.75	206	21303	1.62	1.40–1.85	111	12746	1.58	1.30–1.89	391	39339	1.69	1.52–1.86
Blood	C81–85, C88, C90, C91–96	21274	170	15222	1.24	1.05–1.44	498	47703	1.29	1.18–1.42	234	23182	1.65	1.44–1.87	902	86107	1.36	1.27–1.46

Note:

A metachronous second primary cancer was defined as a subsequent primary cancer that occurred during 3 months to 10 years after diagnosis of the first primary cancer, using the suggested rules of the International Association for Research on Cancer (IARC) and the ICD‐O third edition. Person‐years at risk were calculated as the time from 3 months after diagnosis of the first primary cancer until whichever of the following came first: (i) December 31, 2015, (ii) diagnosis of an SPC, (iii) death, or (iv) 10 years after the FPC diagnosis. SIRs were calculated as the ratio of the observed number to the expected number of second primary cancers to compare the risk of developing an SPC to the general population.

Abbreviations: CI, confidence interval; FPC, first primary cancer; SIR, standardized incidence ratio; SPC, second primary cancer.

SIRs for the respective first‐second site combinations are listed in Table S1 (statistically non‐significant SIRs are suppressed). Among males, especially high SIRs were observed for oral/pharyngeal, esophageal, and laryngeal cancers followed by one another, ranging from 3.02 (95% CI, 1.66–5.27) for laryngeal after oral/pharyngeal cancer to 23.44 (95% CI, 20.66–26.56) for oral/pharyngeal after esophageal cancer. Strong associations (i.e. SIR ≥3.0) were also observed for lung after oral/pharyngeal cancer (SIR = 3.81 [95% CI, 3.28–4.38]); lung after laryngeal cancer (SIR = 4.20 [95% CI, 3.54–4.97]); thyroid after lung cancer (SIR = 3.19 [95% CI, 2.01–5.00]); thyroid after kidney/urinary tract/bladder cancer (SIR = 4.13 [95% CI, 2.62–6.37]); and kidney/urinary tract/bladder after thyroid cancer (SIR = 3.35 [95% CI, 2.02–5.30]) in males. Decreased SIRs were seen for prostate after liver cancer (SIR = 0.75 [95% CI, 0.60–0.92]) and liver after prostate cancer (SIR = 0.72 [95% CI, 0.62–0.85]). Similarly, in females, SIRs were especially high for esophageal after oral/pharyngeal cancer (SIR = 37.78 [95% CI, 26.97–50.71]) and oral/pharyngeal after esophageal cancer (SIR = 50.07 [95% CI, 35.28–68.75]). Other sites with high SIRs included lung after oral/pharyngeal cancer (4.31 [95% CI, 3.03–5.86]); stomach after esophageal cancer (4.30 [95% CI, 2.89–6.69]); lung after esophageal cancer (3.98 [95% CI, 2.48–6.02]); oral/pharyngeal after liver cancer (3.40 [95% CI, 1.79–5.67]); oral/pharyngeal after blood cancer (3.67 [95% CI, 2.02–6.13]); and esophageal after stomach cancer (3.53 [95% CI, 2.40–5.01]) in females. SIRs of developing subsequent thyroid cancer were also high after breast (3.15 [95% CI, 2.65–3.76]), uterus (3.55 [95% CI, 2.52–4.75]), ovary (3.58 [95% CI, 2.00–6.06]), and kidney/urinary tract/bladder cancers (5.20 [95% CI, 3.36–7.99]).

**FIGURE 2:**
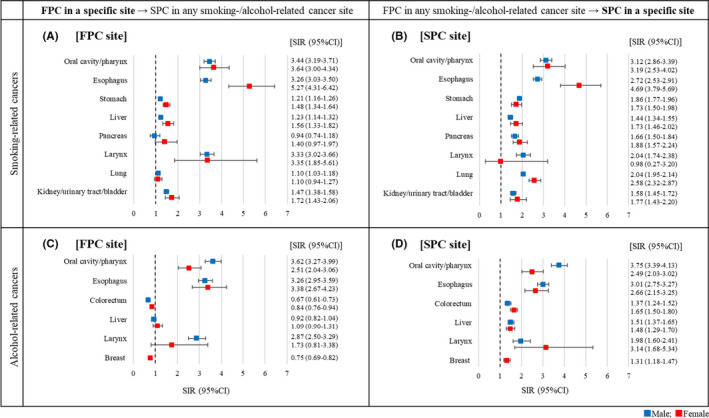
Standardized incidence ratios of developing smoking‐/alcohol‐related metachronous second primary cancers among survivors of smoking‐/alcohol‐related cancers, Osaka Cancer Registry, Japan, 2000–2015. FPC, first primary cancer; SPC, second primary cancer; SIR, standardized incidence ratio; CI, confidence interval. Note: A metachronous second primary cancer was defined as a subsequent primary cancer that occurred during 3 months to 10 years after diagnosis of the first primary cancer. SIRs were calculated as the ratio of the observed number to the expected number of second primary cancers to compare the risk of developing an SPC to the general population

Figure 2 presents the SIRs of developing any smoking‐ or alcohol‐related SPCs for each of the specific FPC sites (panels A & C) and the SIRs of developing SPCs in specific sites after any smoking‐ or alcohol‐related FPCs (panels B & D). Among survivors of the eight respective smoking‐related cancers (Figure 2A), risks of developing SPCs in any of the smoking‐related sites were significantly higher than the general population except among survivors of pancreatic cancer in both sexes and female survivors of lung cancer. SIRs ranged from 1.10 (95% CI, 1.03–1.18) in male lung cancer survivors to 5.27 (95% CI, 4.31–6.42) in female esophageal cancer survivors. Among survivors of any smoking‐related cancers (Figure 2B), risks of developing smoking‐related SPC in a specific site were significantly high for all sites except laryngeal cancers in female. Elevated SIRs ranged from 1.44 (95% CI, 1.34–1.55) for liver cancers in males to 4.69 (95% CI, 3.79–5.69) for esophageal cancers in females. Among survivors of the six respective alcohol‐related cancers (Figure 2C), risks of developing any alcohol‐related SPCs were significantly higher than the general population among survivors of oral/pharyngeal and esophageal cancers in both sexes and male survivors of laryngeal cancer. Elevated SIRs ranged from 2.51 (95% CI, 2.04–3.06) among female oral/pharyngeal cancer survivors to 3.62 (95% CI, 3.27–3.99) among male oral/pharyngeal cancer survivors. Decreased SIRs were observed among male and female colorectum cancer survivors (0.67 [95% CI, 0.61–0.73] and 0.84 [95% CI, 0.76–0.94], respectively) and female breast cancer survivors (0.75 [95% CI, 0.69–0.82]). Among survivors of any alcohol‐related cancers (Figure 2D), risks of developing an alcohol‐related SPC in a specific site were significantly higher for all sites, with SIRs ranging from 1.31 (95% CI, 1.18–1.47) for breast cancer in females to 3.75 (95% CI, 3.39–4.13) for oral/pharyngeal cancer in males.

## 4 DISCUSSION

During 2000–2015, 5.8% of all cancer survivors developed an SPC within 10 years of first diagnosis, showing an increase by 2.0 percentage points since our previous assessment (3.8% during 1985–2005).^4^ Our findings provide clinicians with a benchmark to estimate the probability of cancer survivors developing another cancer. Male survivors had a 38% higher risk, and female survivors had a 44% higher risk of developing SPCs than the general population. Cumulative incidence increased with age and was as high as nearly 20% in the most dominant age group (70–79 years). Given that the elderly represent the majority of overall cancer survivors, developing an SPC is not an uncommon event. During the 10‐year follow‐up, SIRs were highest in 5–10 years after the FPC diagnosis in most of the sex‐age subgroups and the assessed cancer sites, likely due to the accumulated exposure to risk factors over time.^1^, ^5^, ^11^ While elevated risks were observed for 15 of the 16 FPC sites, especially high risks were seen for certain combinations of first‐second cancer sites involving oral cavity/pharynx, esophagus, and larynx, indicating the role of behavioral risk factors such as tobacco smoking and alcohol intake.^2^, ^4^, ^9^, ^11^

We observed elevated SIRs across all sex‐age groups with the highest estimates in young‐onset survivors. Incidence of cancers is attributable to a number of risk factors including smoking, alcohol intake, unbalanced diet, inherited susceptibilities—and for multiple primaries specifically, effects from the treatment of prior cancers.^1^, ^5^, ^11^ Higher SIRs among young‐onset survivors may be partially explained by differences between younger and older populations in various respects such as genetic predisposition and accumulation of exposure to risk factors.^1^, ^25^ Another potential reason is improved survival in adolescent and young adult (AYA, aged 15–39 years) patients: in Osaka, 5‐year survival of AYA patients has constantly increased to around 80% over the past decades.^26^ Given that young‐onset cancer patients tend to receive more aggressive treatment,^1^, ^27^, ^28^ which may increase the likelihood of experiencing long‐term effects of potentially carcinogenic treatment.

Aligned with previous findings,^4^ we observed strong bidirectional associations between oral cavity/pharynx, esophagus, and larynx, highlighting the role of shared risk factors of these cancers. Smoking and alcohol intake are known risk factors of various cancers, and their multiplicative effects can substantially exceed the risk from either factor alone for some cancer sites.^4^, ^9^, ^21^ When we restricted the analyses to smoking‐ or alcohol‐related cancers, we observed elevated SIRs of developing SPCs compared to those of overall cancer survivors for some cancer sites. These findings suggest the effects of smoking and drinking as risk factors of both first and second primaries and pose a question as to how SPCs can be averted by lifestyle changes. It can often be challenging for cancer survivors to change their behaviors even when they have an intention to do so, likely due to lack of assistance.^21^, ^29^, ^30^, ^31^, ^32^ Our findings underscore the importance of coordinated efforts through counseling to facilitate healthier lifestyle, surveillance to understand behavioral patterns of survivors, and promotion of screening for new malignancies.

We observed decreased SIRs of developing alcohol‐related cancers among male and female colorectal cancer survivors and female breast cancer survivors (Figure 2C). It is unlikely that any FPCs serve as a protective factor for patients from developing SPCs. As colorectal and breast cancers are both common and have relatively good survival (i.e. longer person‐years at risk), large numbers of SPCs were expected among survivors of these cancers. Under the rules to define SPCs in the present study, this might have resulted in the underestimation of SIRs when we restricted the analysis to alcohol‐related cancers.

It is noteworthy that bidirectional association was also observed between liver and prostate cancers with significantly decreased SIRs (<1.0). This may be partially attributable to the difference in socioeconomic characteristics associated with each cancer. A number of studies reported the link between prostate cancer detection and higher socioeconomic status due to better utilization of screening and health care services.^33^, ^34^, ^35^ By contrast, liver cancer is associated with lower socioeconomic status as the risk factors such as obesity, diabetes, alcoholism, smoking, and hepatitis B or C infection are more prevalent in underserved populations.^36^, ^37^, ^38^ The role of socioeconomic disparities in the etiology of SPCs is still understudied. Further research is required to fill this knowledge gap and inform local and national survivorship care strategies.

In many of the sex‐age subgroups and cancer sites assessed, we observed higher SIRs during 2000–2015 compared to those from our previous assessment during 1985–2005.^4^ Similar increasing trends were reported in other high‐income countries.^2^, ^39^ The reasons are unknown but may include changes in treatment modalities: in Japan and globally, use of leukemogenic agents and radiotherapy has been increasing in recent years.^40^, ^41^, ^42^, ^43^ In our study, unidirectional associations were seen between blood cancer and several solid cancers, suggesting later effects of chemotherapy and/or radiotherapy.^1^, ^2^, ^11^, ^42^ Elevated SIRs of developing blood cancer were observed after colorectal and kidney/urinary tract/bladder cancers in both sexes; esophageal and prostate cancers in males; and breast, uterus, and ovary cancers in females, while reverse associations were not significant. Furthermore, higher risks of developing SPCs were observed in close proximity to the FPC sites (e.g. prostate after kidney/urinary tract/bladder cancer; lung after breast cancer; uterus and ovary after kidney/urinary tract/bladder cancer), potentially due to late effects of radiotherapy.^1^, ^2^, ^11^, ^44^ As an increasing number of patients are expected to receive chemotherapy and/or radiotherapy,^43^, ^45^ quantifying the effects of prior treatment is imperative to inform risk‐based long‐term follow‐up care planning.

Our study is subject to several limitations. First, we were unable to rule out the bias that might have been caused by invalid registrations (i.e. DCO registrations) excluded from the analyses. Incompleteness of the data affects estimated incidence rates in the general population and both observed and expected numbers of SPCs, which subsequently leads to overestimation or underestimation of SIRs. However, the DCO rates of the Osaka Cancer Registry substantially improved from 10%–15% in our previous assessments (1960s—early 2000s) to 5.8% in the present study (2005–2014) which satisfied the international standard for completeness (DCO <10%).^46^ Thus, we assume that such bias was kept minimum in this study. Second, we excluded any subsequent primaries that occurred in the same site as the FPC to minimize misclassification of recurrence. This might have led underestimation of the true risk of SPCs that may include multicentric tumors, new primaries at the other side of paired organs, and those with different histology. Third, there is a possibility that detection bias affected the estimates of SIRs even though we excluded synchronous second primaries (occurred within 3 months of first diagnosis) from the analyses. For example, the elevated SIRs of subsequent diagnosis with thyroid cancer might be the result of heightened medical surveillance following the initial cancer diagnosis.^2^, ^4^, ^8^, ^9^, ^47^, ^48^ Fourth, cumulative risks presented in Table 1 may have been underestimated due to incomplete follow‐up of individuals who were diagnosed in recent years (i.e. those diagnosed in/after 2006 not fully followed up for 10 years, and those diagnosed in/after 2011 not fully followed up for 5 years). Fifth, we could not assess the risk of SPCs beyond ten years as ten years is the maximum length of follow‐up in the OCR. Lastly, we were unable to assess the impact of important risk factors of SPCs other than age and sex. The Osaka Cancer Registry and other major population‐based cancer registries do not usually collect information on patients’ socioeconomic or behavioral characteristics, comorbidity history, and follow‐up care. Thus, the heterogeneity of such factors between cancer survivors and the general population is unknown.

## 5 CONCLUSION

Cancer survivors were at excess risk of developing SPCs compared to the general population. Both cumulative and relative risks of SPCs showed constant increases since the 1960s. Given the growing importance of survivorship care, continued surveillance is warranted to understand the risk patterns and trends of SPCs and inform the efforts to prevent new cancers among survivors.

## CONFLICTS OF INTEREST

Authors have no potential conflict of interest to declare.

## AUTHOR CONTRIBUTION

Satomi Odani designed the study, performed data analyses, and drafted the manuscript. Takahiro Tabuchi helped conceptualize the study and perform data analyses. All authors contributed to data aquisition and interpretation and critically reviewed and revised the manuscript.

## Supporting information

Table S1

## Data Availability

The OCR data are not publicly accessible and are available only on request due to privacy and ethical restrictions.
