# Supplementary material for: Incidence and relative risk of metachronous second primary cancers for 16 cancer sites, Osaka, Japan, 2000–2015: Population‐based analysis
Source: Cancer Med. 2021 Nov 29;11(2):507–19. doi: 10.1002/cam4.4457 (PMC8729047; doi:10.1002/cam4.4457)
Supplement: Supplementary file 1 — Table S1 [file CAM4-11-507-s001.docx]

**Supplementary Table 1.** Observed numbers and standardized incidence ratios of developing a second primary cancer for each first-second site combination by sex, Osaka Cancer Registry, Japan, 2000-2015

| FPC site | SPC site | Observed  number of  SPC | Person-years | SIR | 95%CI |
| --- | --- | --- | --- | --- | --- |
| **Male** |  |  |  |  |  |
| Any | Oral cavity/pharynx | 704 | 847526 | 2.19 | 2.03-2.36 |
| Any | Esophagus | 1117 | 848301 | 2.02 | 1.90-2.14 |
| Any | Stomach | 3315 | 678210 | 1.67 | 1.61-1.73 |
| Any | Colorectum | 2023 | 700163 | 1.25 | 1.19-1.31 |
| Any | Liver | 1422 | 809396 | 1.27 | 1.21-1.35 |
| Any | Gallbladder | 378 | 866334 | 1.51 | 1.35-1.68 |
| Any | Pancreas | 697 | 866030 | 1.52 | 1.41-1.65 |
| Any | Larynx | 243 | 860018 | 1.63 | 1.42-1.85 |
| Any | Lung | 3329 | 791626 | 1.73 | 1.67-1.79 |
| Any | Prostate | 2167 | 729699 | 1.73 | 1.65-1.80 |
| Any | Kidney/urinary tract/bladder | 1138 | 804641 | 1.52 | 1.43-1.62 |
| Any | Thyroid | 133 | 865723 | 2.16 | 1.82-2.57 |
| Any | Blood | 837 | 832344 | 1.53 | 1.43-1.65 |
| Oral cavity/pharynx | Esophagus | 231 | 28007 | 15.77 | 13.86-17.87 |
| Oral cavity/pharynx | Stomach | 137 | 27837 | 2.16 | 1.81-2.56 |
| Oral cavity/pharynx | Colorectum | 80 | 27715 | 1.63 | 1.30-2.02 |
| Oral cavity/pharynx | Liver | 61 | 27643 | 2.14 | 1.63-2.72 |
| Oral cavity/pharynx | Gallbladder | 13 | 27487 | 2.13 | 1.14-3.63 |
| Oral cavity/pharynx | Pancreas | 21 | 27497 | 1.96 | 1.24-2.94 |
| Oral cavity/pharynx | Larynx | 11 | 27505 | 3.02 | 1.66-5.27 |
| Oral cavity/pharynx | Lung | 185 | 27812 | 3.81 | 3.28-4.38 |
| Oral cavity/pharynx | Prostate | 52 | 27639 | 1.41 | 1.05-1.85 |
| Oral cavity/pharynx | Blood | 36 | 27520 | 2.32 | 1.64-3.29 |
| Esophagus | Oral cavity/pharynx | 239 | 27203 | 23.44 | 20.66-26.56 |
| Esophagus | Stomach | 210 | 27202 | 2.82 | 2.45-3.24 |
| Esophagus | Colorectum | 78 | 26885 | 1.30 | 1.02-1.63 |
| Esophagus | Liver | 55 | 26805 | 1.58 | 1.18-2.05 |
| Esophagus | Gallbladder | 17 | 26727 | 2.28 | 1.31-3.67 |
| Esophagus | Pancreas | 23 | 26734 | 1.80 | 1.19-2.71 |
| Esophagus | Larynx | 39 | 26820 | 8.99 | 6.53-12.18 |
| Esophagus | Lung | 140 | 27017 | 2.44 | 2.05-2.86 |
| Esophagus | Prostate | 62 | 26849 | 1.38 | 1.05-1.77 |
| Esophagus | Kidney/urinary tract/bladder | 48 | 26794 | 2.10 | 1.54-2.78 |
| Esophagus | Blood | 30 | 26757 | 2.02 | 1.39-2.82 |
| Stomach | Oral cavity/pharynx | 119 | 197093 | 1.55 | 1.29-1.86 |
| Stomach | Esophagus | 273 | 197436 | 2.12 | 1.87-2.39 |
| Stomach | Colorectum | 549 | 198374 | 1.26 | 1.15-1.37 |
| Stomach | Liver | 402 | 197723 | 1.50 | 1.35-1.67 |
| Stomach | Gallbladder | 80 | 196949 | 1.48 | 1.16-1.85 |
| Stomach | Pancreas | 168 | 196964 | 1.67 | 1.42-1.96 |
| Stomach | Larynx | 49 | 196946 | 1.45 | 1.06-1.91 |
| Stomach | Lung | 936 | 198543 | 1.97 | 1.84-2.11 |
| Stomach | Prostate | 672 | 198845 | 1.91 | 1.76-2.07 |
| Stomach | Kidney/urinary tract/bladder | 285 | 197561 | 1.65 | 1.45-1.86 |
| Stomach | Blood | 196 | 197165 | 1.56 | 1.34-1.80 |
| Colorectum | Esophagus | 165 | 175170 | 1.43 | 1.21-1.66 |
| Colorectum | Stomach | 818 | 177073 | 1.64 | 1.53-1.77 |
| Colorectum | Liver | 303 | 175494 | 1.33 | 1.18-1.49 |
| Colorectum | Gallbladder | 77 | 174948 | 1.61 | 1.25-2.02 |
| Colorectum | Pancreas | 120 | 174967 | 1.30 | 1.06-1.57 |
| Colorectum | Lung | 648 | 176175 | 1.55 | 1.43-1.68 |
| Colorectum | Prostate | 554 | 176484 | 1.79 | 1.63-1.95 |
| Colorectum | Kidney/urinary tract/bladder | 214 | 175519 | 1.35 | 1.17-1.56 |
| Colorectum | Thyroid | 27 | 174949 | 2.16 | 1.43-3.10 |
| Colorectum | Blood | 156 | 175114 | 1.43 | 1.21-1.68 |
| Liver | Oral cavity/pharynx | 42 | 65679 | 1.64 | 1.20-2.22 |
| Liver | Esophagus | 68 | 65720 | 1.52 | 1.18-1.93 |
| Liver | Stomach | 360 | 66527 | 1.77 | 1.58-1.97 |
| Liver | Colorectum | 188 | 66020 | 1.21 | 1.03-1.40 |
| Liver | Gallbladder | 29 | 65631 | 1.53 | 1.05-2.24 |
| Liver | Prostate | 101 | 65867 | 0.75 | 0.60-0.92 |
| Liver | Kidney/urinary tract/bladder | 87 | 65819 | 1.34 | 1.05-1.66 |
| Liver | Blood | 82 | 65718 | 1.80 | 1.41-2.26 |
| Gallbladder | Pancreas | 10 | 8681 | 2.01 | 1.00-3.83 |
| Gallbladder | Kidney/urinary tract/bladder | 17 | 8705 | 2.19 | 1.27-3.43 |
| Larynx | Oral cavity/pharynx | 38 | 15069 | 6.78 | 4.94-9.28 |
| Larynx | Esophagus | 66 | 15160 | 6.98 | 5.46-8.83 |
| Larynx | Stomach | 115 | 15373 | 2.71 | 2.24-3.28 |
| Larynx | Colorectum | 58 | 15147 | 1.78 | 1.35-2.30 |
| Larynx | Liver | 47 | 15092 | 2.36 | 1.74-3.17 |
| Larynx | Lung | 146 | 15283 | 4.20 | 3.54-4.97 |
| Larynx | Prostate | 46 | 15137 | 1.90 | 1.39-2.51 |
| Larynx | Kidney/urinary tract/bladder | 27 | 15070 | 2.11 | 1.43-3.11 |
| Lung | Oral cavity/pharynx | 56 | 83516 | 1.73 | 1.32-2.25 |
| Lung | Esophagus | 90 | 83526 | 1.60 | 1.28-1.96 |
| Lung | Stomach | 392 | 84134 | 1.57 | 1.41-1.74 |
| Lung | Gallbladder | 43 | 83417 | 1.73 | 1.23-2.35 |
| Lung | Pancreas | 78 | 83457 | 1.71 | 1.33-2.15 |
| Lung | Larynx | 33 | 83464 | 2.26 | 1.57-3.19 |
| Lung | Prostate | 216 | 83994 | 1.36 | 1.18-1.56 |
| Lung | Kidney/urinary tract/bladder | 118 | 83608 | 1.48 | 1.21-1.78 |
| Lung | Thyroid | 19 | 83441 | 3.19 | 2.01-5.00 |
| Lung | Blood | 77 | 83526 | 1.42 | 1.11-1.78 |
| Prostate | Stomach | 751 | 147066 | 1.40 | 1.29-1.51 |
| Prostate | Colorectum | 471 | 146601 | 1.12 | 1.01-1.23 |
| Prostate | Liver | 195 | 145720 | 0.72 | 0.62-0.85 |
| Prostate | Pancreas | 129 | 145446 | 1.38 | 1.13-1.65 |
| Prostate | Lung | 558 | 146275 | 1.21 | 1.10-1.33 |
| Prostate | Kidney/urinary tract/bladder | 251 | 145915 | 1.47 | 1.28-1.68 |
| Prostate | Thyroid | 24 | 145363 | 2.03 | 1.27-3.01 |
| Prostate | Blood | 155 | 145595 | 1.36 | 1.14-1.61 |
| Kidney/urinary tract/bladder | Oral cavity/pharynx | 45 | 70477 | 1.64 | 1.19-2.20 |
| Kidney/urinary tract/bladder | Esophagus | 72 | 70493 | 1.56 | 1.21-1.97 |
| Kidney/urinary tract/bladder | Stomach | 329 | 71193 | 1.66 | 1.48-1.86 |
| Kidney/urinary tract/bladder | Colorectum | 228 | 70946 | 1.45 | 1.26-1.66 |
| Kidney/urinary tract/bladder | Liver | 119 | 70608 | 1.23 | 1.01-1.50 |
| Kidney/urinary tract/bladder | Gallbladder | 35 | 70414 | 1.80 | 1.22-2.50 |
| Kidney/urinary tract/bladder | Pancreas | 65 | 70443 | 1.98 | 1.54-2.54 |
| Kidney/urinary tract/bladder | Lung | 315 | 70959 | 1.93 | 1.71-2.16 |
| Kidney/urinary tract/bladder | Prostate | 331 | 71452 | 2.80 | 2.49-3.12 |
| Kidney/urinary tract/bladder | Thyroid | 19 | 70405 | 4.13 | 2.62-6.37 |
| Kidney/urinary tract/bladder | Blood | 72 | 70511 | 1.69 | 1.32-2.15 |
| Thyroid | Stomach | 26 | 9355 | 1.55 | 1.04-2.30 |
| Thyroid | Prostate | 25 | 9358 | 2.74 | 1.79-4.01 |
| Thyroid | Kidney/urinary tract/bladder | 17 | 9319 | 3.35 | 2.02-5.30 |
| Blood | Oral cavity/pharynx | 27 | 42705 | 1.95 | 1.28-2.83 |
| Blood | Stomach | 127 | 42954 | 1.34 | 1.10-1.60 |
| Blood | Liver | 77 | 42808 | 1.75 | 1.38-2.20 |
| Blood | Lung | 129 | 42879 | 1.67 | 1.39-2.00 |
| **Female** |  |  |  |  |  |
| Any | Oral cavity/pharynx | 152 | 772794 | 2.1 | 1.78-2.45 |
| Any | Esophagus | 147 | 779070 | 2.37 | 2.01-2.77 |
| Any | Stomach | 865 | 698550 | 1.69 | 1.57-1.80 |
| Any | Colorectum | 1027 | 660524 | 1.62 | 1.53-1.73 |
| Any | Liver | 421 | 756645 | 1.51 | 1.36-1.66 |
| Any | Gallbladder | 183 | 778476 | 1.56 | 1.33-1.81 |
| Any | Pancreas | 347 | 778429 | 1.76 | 1.58-1.96 |
| Any | Larynx | 16 | 784261 | 2.26 | 1.37-3.70 |
| Any | Lung | 1091 | 735197 | 2.34 | 2.20-2.49 |
| Any | Breast | 904 | 518855 | 1.3 | 1.22-1.38 |
| Any | Uterus | 403 | 706908 | 1.34 | 1.22-1.48 |
| Any | Ovary | 184 | 760813 | 1.39 | 1.20-1.59 |
| Any | Kidney/urinary tract/bladder | 273 | 762940 | 1.83 | 1.62-2.06 |
| Any | Thyroid | 340 | 755576 | 2.93 | 2.63-3.25 |
| Any | Blood | 463 | 748120 | 1.84 | 1.68-2.02 |
| Oral cavity/pharynx | Esophagus | 38 | 12825 | 37.78 | 26.97-50.71 |
| Oral cavity/pharynx | Liver | 13 | 12750 | 2.58 | 1.42-4.30 |
| Oral cavity/pharynx | Lung | 36 | 12800 | 4.31 | 3.03-5.86 |
| Esophagus | Oral cavity/pharynx | 32 | 6535 | 50.07 | 35.28-68.75 |
| Esophagus | Stomach | 24 | 6497 | 4.30 | 2.89-6.39 |
| Esophagus | Colorectum | 18 | 6485 | 2.27 | 1.41-3.60 |
| Esophagus | Lung | 20 | 6475 | 3.98 | 2.48-6.02 |
| Stomach | Esophagus | 30 | 87035 | 3.53 | 2.40-5.01 |
| Stomach | Colorectum | 200 | 87516 | 1.80 | 1.56-2.08 |
| Stomach | Liver | 89 | 87176 | 2.02 | 1.62-2.50 |
| Stomach | Pancreas | 55 | 87020 | 1.95 | 1.46-2.54 |
| Stomach | Lung | 174 | 87401 | 2.39 | 2.03-2.77 |
| Stomach | Breast | 157 | 87544 | 1.32 | 1.12-1.54 |
| Stomach | Kidney/urinary tract/bladder | 41 | 87059 | 1.86 | 1.32-2.50 |
| Stomach | Thyroid | 37 | 87113 | 2.55 | 1.82-3.46 |
| Stomach | Blood | 52 | 87076 | 1.50 | 1.12-1.96 |
| Colorectum | Stomach | 219 | 125668 | 1.89 | 1.65-2.16 |
| Colorectum | Liver | 84 | 125187 | 1.33 | 1.06-1.65 |
| Colorectum | Gallbladder | 43 | 125065 | 1.72 | 1.22-2.31 |
| Colorectum | Pancreas | 71 | 125084 | 1.77 | 1.38-2.22 |
| Colorectum | Lung | 195 | 125517 | 1.88 | 1.63-2.17 |
| Colorectum | Breast | 238 | 125838 | 1.34 | 1.18-1.52 |
| Colorectum | Kidney/urinary tract/bladder | 68 | 125211 | 2.19 | 1.69-2.76 |
| Colorectum | Thyroid | 57 | 125239 | 2.62 | 2.00-3.37 |
| Colorectum | Blood | 74 | 125163 | 1.40 | 1.10-1.77 |
| Liver | Oral cavity/pharynx | 14 | 28899 | 3.40 | 1.79-5.67 |
| Liver | Stomach | 66 | 29039 | 1.97 | 1.52-2.53 |
| Liver | Lung | 56 | 28949 | 1.77 | 1.31-2.32 |
| Liver | Blood | 31 | 28912 | 2.15 | 1.42-3.04 |
| Gallbladder | Colorectum | 17 | 7078 | 1.71 | 1.00-2.72 |
| Pancreas | Lung | 17 | 7119 | 2.83 | 1.69-4.43 |
| Lung | Esophagus | 14 | 50337 | 2.84 | 1.55-4.51 |
| Lung | Stomach | 72 | 50502 | 1.48 | 1.15-1.86 |
| Lung | Pancreas | 34 | 50352 | 1.90 | 1.33-2.71 |
| Lung | Thyroid | 25 | 50402 | 2.83 | 1.83-4.04 |
| Lung | Blood | 42 | 50419 | 1.94 | 1.39-2.61 |
| Breast | Oral cavity/pharynx | 33 | 266756 | 1.51 | 1.04-2.08 |
| Breast | Stomach | 261 | 267292 | 1.65 | 1.45-1.86 |
| Breast | Colorectum | 360 | 267812 | 1.69 | 1.53-1.88 |
| Breast | Liver | 105 | 266932 | 1.49 | 1.22-1.79 |
| Breast | Gallbladder | 63 | 266733 | 2.17 | 1.69-2.79 |
| Breast | Pancreas | 94 | 266776 | 1.86 | 1.51-2.27 |
| Breast | Lung | 299 | 267420 | 2.27 | 2.02-2.54 |
| Breast | Uterus | 192 | 267201 | 1.69 | 1.47-1.94 |
| Breast | Ovary | 72 | 266904 | 1.53 | 1.22-1.91 |
| Breast | Kidney/urinary tract/bladder | 67 | 266898 | 1.65 | 1.28-2.07 |
| Breast | Thyroid | 118 | 267165 | 3.15 | 2.65-3.76 |
| Breast | Blood | 146 | 266949 | 2.01 | 1.70-2.35 |
| Uterus | Stomach | 61 | 78751 | 1.46 | 1.12-1.86 |
| Uterus | Colorectum | 103 | 78927 | 1.93 | 1.59-2.32 |
| Uterus | Lung | 105 | 78839 | 3.15 | 2.59-3.78 |
| Uterus | Breast | 145 | 79030 | 1.50 | 1.27-1.74 |
| Uterus | Kidney/urinary tract/bladder | 21 | 78671 | 1.98 | 1.26-2.99 |
| Uterus | Thyroid | 38 | 78723 | 3.55 | 2.52-4.75 |
| Uterus | Blood | 42 | 78692 | 2.18 | 1.59-2.90 |
| Ovary | Colorectum | 31 | 24790 | 1.76 | 1.23-2.48 |
| Ovary | Lung | 33 | 24808 | 3.11 | 2.15-4.24 |
| Ovary | Thyroid | 12 | 24734 | 3.58 | 2.00-6.06 |
| Ovary | Blood | 14 | 24726 | 2.35 | 1.35-3.77 |
| Kidney/urinary tract/bladder | Stomach | 34 | 22661 | 1.50 | 1.04-2.12 |
| Kidney/urinary tract/bladder | Colorectum | 61 | 22730 | 2.11 | 1.62-2.72 |
| Kidney/urinary tract/bladder | Lung | 56 | 22690 | 2.93 | 2.21-3.80 |
| Kidney/urinary tract/bladder | Breast | 43 | 22682 | 1.39 | 1.02-1.85 |
| Kidney/urinary tract/bladder | Uterus | 23 | 22648 | 2.39 | 1.53-3.49 |
| Kidney/urinary tract/bladder | Ovary | 10 | 22583 | 2.60 | 1.35-4.51 |
| Kidney/urinary tract/bladder | Thyroid | 19 | 22638 | 5.20 | 3.36-7.99 |
| Kidney/urinary tract/bladder | Blood | 21 | 22613 | 2.23 | 1.39-3.37 |
| Thyroid | Stomach | 29 | 30024 | 1.50 | 1.02-2.15 |
| Thyroid | Colorectum | 36 | 30055 | 1.43 | 1.02-1.97 |
| Thyroid | Pancreas | 16 | 29960 | 2.66 | 1.54-4.16 |
| Thyroid | Lung | 47 | 30068 | 2.94 | 2.20-3.91 |
| Thyroid | Breast | 61 | 30167 | 1.63 | 1.27-2.09 |
| Thyroid | Ovary | 11 | 29972 | 2.19 | 1.22-3.88 |
| Thyroid | Blood | 19 | 29975 | 2.24 | 1.35-3.37 |
| Blood | Oral cavity/pharynx | 13 | 37419 | 3.67 | 2.02-6.13 |
| Blood | Stomach | 48 | 37512 | 1.61 | 1.17-2.13 |
| Blood | Liver | 25 | 37427 | 1.69 | 1.12-2.51 |
| Blood | Lung | 39 | 37472 | 1.59 | 1.13-2.15 |
| Blood | Thyroid | 13 | 37426 | 2.33 | 1.32-3.83 |

**Abbreviations:** FPC = first primary cancer; SPC = second primary cancer; SIR = standardized incidence ratio; CI = confidence interval.

**Note:** Statistically non-significant SIRs are not presented. A metachronous second primary cancer was defined as a subsequent primary cancer that occurred during 3 months to 10 years after diagnosis of the first primary cancer. Person-years at risk were calculated as the time from 3 months after diagnosis of the first primary cancer until whichever of the following came first: (i) December 31^st^, 2015, (ii) diagnosis of an SPC, (iii) death, or (iv) 10 years after the FPC diagnosis. SIRs were calculated as the ratio of the observed number to the expected number of second primary cancers to compare the risk of developing an SPC to the general population.
